# Analysis of the Chemotherapy-Free Interval following Image-Guided Ablation in Sarcoma Patients

**DOI:** 10.1155/2020/3852420

**Published:** 2020-02-14

**Authors:** Charles Sutton, Yachao Zhang, DaeHee Kim, Hooman Yarmohammadi, Etay Ziv, Franz E. Boas, Constantinos T. Sofocleous, William D. Tap, Sandra P. D'Angelo, Joseph P. Erinjeri

**Affiliations:** ^1^Interventional Radiology Service, Department of Radiology, Memorial Sloan Kettering Cancer Center, New York, NY, USA; ^2^Department of Medicine, Memorial Sloan Kettering Cancer Center, New York, NY, USA; ^3^Weill Cornell Medical College, New York, NY, USA

## Abstract

One way to enhance quality of life for patients with metastatic sarcoma is to maximize time off chemotherapy—a chemotherapy-free interval. While image-guided ablation of sarcoma metastases may reduce the need for chemotherapy, it remains unknown how long ablation could extend the chemotherapy-free interval. The purpose of our study was to determine the chemotherapy-free interval in comparison to overall survival and progression-free survival in sarcoma patients who undergo ablation procedures. An IRB-approved, single institution, HIPAA compliant database was queried for sarcoma patients who underwent image-guided ablation procedures between 2007 and 2018. Patient demographics, histologic subtype, and other clinical characteristics were recorded. Kaplan-Meier analysis was performed to compute median overall survival, median progression-free survival (local and distant), and the median chemotherapy-free interval (systemic and cytotoxic) after ablation. Univariate and multivariate analyses were performed using the log-rank test and Cox proportional-hazards model, respectively. A total of 100 sarcoma patients were included in the analysis. The most common histologic subtype was leiomyosarcoma (38%). Median overall survival after ablation of sarcoma metastases was 52.4 months (95% CI: 46.9–64.0 months). The median systemic chemotherapy-free interval following ablation of sarcoma metastases was 14.7 months (95% CI: 8.6–34.3 months). The median cytotoxic chemotherapy-free interval following ablation of sarcoma metastases was 81.3 months (95% CI: 34.3-median not reached). In conclusion, ablation of sarcoma metastases can provide an extended systemic chemotherapy-free interval of greater than 1 year. Ablation of sarcoma metastases may improve patient quality of life by extending the chemotherapy-free interval.

## 1. Introduction

Most sarcoma patients are diagnosed with localized disease, which can often be cured through a combination of surgery and radiation therapy. However, some sarcoma patients, particularly those with large primary tumours, presenting initially with localized disease may ultimately experience disease progression at metastatic sites [1, 2]. Although not curative, cytotoxic chemotherapy continues to be the mainstay of treatment for metastatic soft tissue sarcomas [3]. For most soft tissue sarcomas, doxorubicin or gemcitabine and docetaxel is the standard 1st line regimen, with pazopanib as a viable 2nd line option [4, 5]. While chemotherapy has traditionally been used for patients with metastatic sarcoma, classical regimens typically do not provide a survival benefit [6]. Furthermore, these agents are associated with adverse effects, such as cardiomyopathy (doxorubicin) and vascular toxicity (gemcitabine) [7, 8]. In cancer patients who cannot be cured, treatment should focus on creating both increased quantity and quality of life [9]. One strategy to enhance quality of life for patients with incurable cancer is to maximize time without toxic effects, which can be achieved by providing time off chemotherapy—a chemotherapy-free interval [10].

For patients that present with primary sarcoma, surgery remains the only curative treatment option [11]. While current treatments for patients with metastatic sarcoma are not curative, one option, image-guided ablation of sarcoma metastases, can benefit these patients by providing an extended chemotherapy-free interval, improving quality of life. Multiple studies have identified the benefits of ablation as a therapeutic option in the management of patients with sarcoma [12–16]. Ablation is minimally invasive, lacks treatment-specific toxicity, and provides a lower-morbidity treatment option compared to surgical resection [17, 18]. For patients with metastatic sarcoma, while image-guided ablation may extend the chemotherapy-free interval, it remains unknown how long ablation could extend the chemotherapy-free interval. We hypothesize that image-guided ablation will provide a significant chemotherapy-free interval in patients with metastatic sarcoma. The purpose of our study was to determine the chemotherapy-free interval in comparison to overall survival and progression-free survival in sarcoma patients who undergo ablation procedures.

## 2. Materials and Methods

### 2.1. Ablation Procedures

An IRB-approved, single institution, HIPAA compliant database was queried for sarcoma patients who underwent image-guided ablation procedures between 2007 and 2018. All patients over the 11-year period were included in the analysis except for those patients who underwent image-guided ablation for palliative purposes. Patient demographics, tumour size, tumour grade, histologic subtype, whether the patient had failed previous chemotherapy, and the disease-free interval prior to ablation were investigated. Age was defined as the interval from the patient's date of birth to the date of the patient's initial ablation procedure for sarcoma (ablation date). Tumour size was defined as the largest measured transaxial length of the treated tumour; in cases where 2 or more tumours were treated, tumour size was defined as the sum of the largest measured transaxial lengths of the treated tumours. Index tumour grade was defined as the histologic grade of the primary tumour. Failure of previous chemotherapy was defined as having received any systemic chemotherapy (both cytotoxic and noncytotoxic) prior to the ablation date. Disease-free interval was defined as the interval from the date of the patient's most recent surgical resection for sarcoma prior to the ablation date (conditional upon initial follow-up imaging not demonstrating evidence of residual or recurrent disease) to the date of the first imaging study thereafter that demonstrated evidence of recurrent disease.

### 2.2. Study Assessments

Median overall survival, median progression-free survival (local and distant), and the median chemotherapy-free interval (systemic and cytotoxic) after ablation were computed using Kaplan-Meier analysis. Overall survival was defined as the interval from the ablation date to the date of death. Patients who were not confirmed to have died at the end of the study were censored at the date of last contact. Local progression-free survival was defined as the interval from the ablation date to the date of the first imaging study thereafter that demonstrated evidence of recurrent disease in the ablation cavity or death. Distant progression-free survival was defined as the interval from the ablation date to the date of the first imaging study thereafter that demonstrated evidence of disease elsewhere or death. Patients who were alive and had not experienced local and/or distant progression at the end of the study were censored at the date of the last available surveillance imaging study. The systemic chemotherapy-free interval was defined as the interval from the ablation date to the date of the earliest subsequent documented administration of systemic chemotherapy (both cytotoxic and noncytotoxic). The cytotoxic chemotherapy-free interval was defined as the interval from the ablation date to the date of the earliest subsequent documented administration of systemic chemotherapy that was cytotoxic. Patients whose systemic and/or cytotoxic chemotherapy-free interval was not concluded at the end of the study were censored at the date of last contact.

### 2.3. Statistical Analysis

Univariate and multivariate analyses were performed using the log-rank test and Cox proportional-hazards model, respectively. The end points for both the univariate and multivariate analyses were overall survival, the systemic chemotherapy-free interval, and the cytotoxic chemotherapy-free interval. The log-rank test was used to analyse the effect of each prognostic factor on the event rate; factors with *p* < 0.05 on log-rank were said to be predictive of the event rate. The Cox proportional-hazards model was used to analyse the independent prognostic value of factors; factors with *p* < 0.1 on log-rank were entered into the multivariate analysis. For all factors included in the multivariate analysis, hazard ratios with confidence intervals were calculated. Factors with *p* < 0.05 on multivariate analysis were said to be significantly associated with the time of an event. All statistical analyses were performed using R version 3.5.1 [19].

## 3. Results

A total of 100 sarcoma patients were included in the analysis.  Table 1 lists the demographic and clinical characteristics of all patients in the study population. There were nearly equal numbers of men and women (45 vs. 55) and the mean age was 59 ± 15 years. 79% of patients had a tumour size ≤3 cm and the mean tumour size was 2.2 ± 1.4 cm. 61% of patients had a high-grade primary sarcoma. The most common histologic subtype was leiomyosarcoma (38%), followed by GIST (14%), liposarcoma (12%), and chondrosarcoma (7%). 52% of patients had failed previous systemic chemotherapy (both cytotoxic and noncytotoxic). 68% of patients had a disease-free interval <12 months (this group includes patients with no disease-free interval). 56% of patients (56/100) received some form of systemic therapy (cytotoxic and/or noncytotoxic), while 36% of patients (36/100) received some form of cytotoxic chemotherapy.

**Figure 1:**
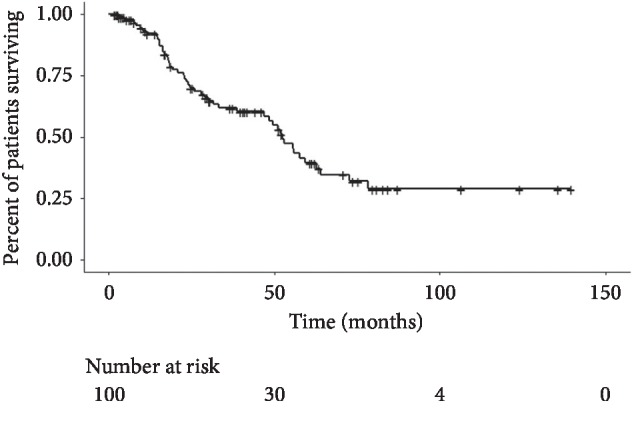
Overall survival. Median overall survival was 52.4 months (95% CI: 46.9–64.0 months) after ablation of sarcoma metastases.

**Figure 2:**
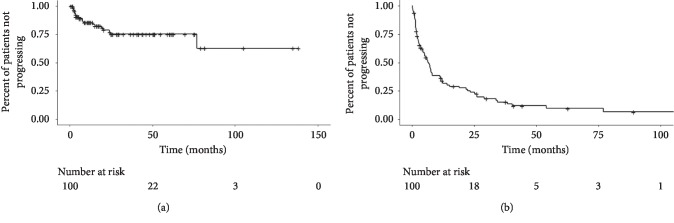
Progression-free survival. (a) Median local progression-free survival after ablation of sarcoma metastases was not reached. (b) Median distant progression-free survival was 6.7 months (95% CI: 4.4–11.2 months) after ablation of sarcoma metastases.

**Figure 3:**
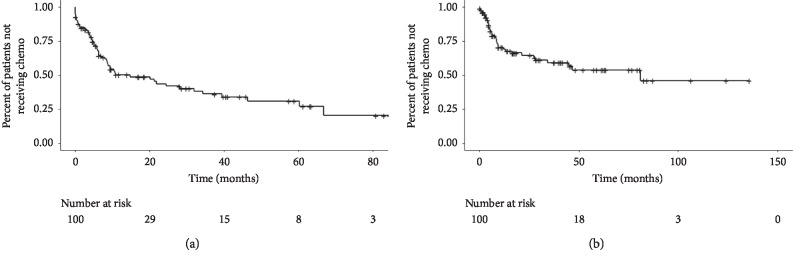
Chemotherapy-free interval. (a) The median systemic chemotherapy-free interval following ablation of sarcoma metastases was 14.7 months (95% CI: 8.6–34.3 months). (b) The median cytotoxic chemotherapy-free interval following ablation of sarcoma metastases was 81.3 months (95% CI: 34.3—median not reached).

Kaplan–Meier curves for overall survival, progression-free survival (local and distant) and the chemotherapy-free interval (systemic and cytotoxic) are shown in Figures 1–3, respectively. Median overall survival was 52.4 months (95% CI: 46.9–64.0 months) after ablation of sarcoma metastases. Median local progression-free survival after ablation of sarcoma metastases was not reached. Median distant progression-free survival was 6.7 months (95% CI: 4.4–11.2 months) after ablation of sarcoma metastases. The median systemic (cytotoxic or noncytotoxic) chemotherapy-free interval following ablation of sarcoma metastases was 14.7 months (95% CI: 8.6–34.3 months). The median cytotoxic chemotherapy-free interval following ablation of sarcoma metastases was 81.3 months (95% CI: 34.3—median not reached).

Results from the univariate and multivariate analyses of overall survival are shown in Table 2. On univariate analysis, tumour size and failure of previous chemotherapy were predictive of overall survival. Patients with a tumour size ≤3 cm had a longer median overall survival compared to patients with a tumour size >3 cm (53.1 vs. 31.6 months, *p*=0.033). Patients who had not failed previous chemotherapy had a longer median overall survival compared to patients who had failed previous chemotherapy (62.6 vs. 33.2 months, *p*=0.027). On multivariate analysis, neither tumour size (*p*=0.1159) nor failure of previous chemotherapy (*p*=0.0749) was significantly associated with time of death.

Results from the univariate and multivariate analyses of the systemic (cytotoxic or noncytotoxic) chemotherapy-free interval are shown in Table 3. On univariate analysis, histologic subtype and failure of previous chemotherapy were predictive of the systemic chemotherapy-free interval. Most notably, patients with liposarcoma had a longer median systemic chemotherapy-free interval compared to patients with all other histologic subtypes (median not reached vs. 9.5 months, *p*=0.0037). Patients who had not failed previous chemotherapy had a longer median systemic chemotherapy-free interval compared to patients who had failed previous chemotherapy (66.8 vs. 6.1 months, *p* < 0.0001). On multivariate analysis, histologic subtype (*p*=0.758) was not significantly associated with time of administration of systemic chemotherapy (both cytotoxic and noncytotoxic), but failure of previous chemotherapy (*p* < 0.0001) was significantly associated with time of administration of systemic chemotherapy (both cytotoxic and noncytotoxic).

Results from the univariate and multivariate analyses of the cytotoxic chemotherapy-free interval are shown in Table 4. On univariate analysis, histologic subtype and failure of previous chemotherapy were predictive of the cytotoxic chemotherapy-free interval. Most notably, patients with leiomyosarcoma had a shorter median cytotoxic chemotherapy-free interval compared to patients with all other histologic subtypes (28.1 months vs. median not reached, *p*=0.01). Patients who had not failed previous chemotherapy had a longer median cytotoxic chemotherapy-free interval compared to patients who had failed previous chemotherapy (median not reached vs. 12.2 months, *p* < 0.0001). On multivariate analysis, histologic subtype (*p*=0.751) was not significantly associated with time of administration of cytotoxic chemotherapy, but failure of previous chemotherapy (*p* < 0.0001) was significantly associated with time of administration of cytotoxic chemotherapy.

## 4. Discussion

Our results suggest that patients who undergo image-guided ablation of sarcoma metastases are able to remain off systemic chemotherapy (both cytotoxic and noncytotoxic) for approximately 14 months. Despite experiencing postablation recurrence after a median of 6.7 months, repeat ablation allowed patients to remain off systemic chemotherapy (both cytotoxic and noncytotoxic) for an additional 8 months thereafter. While other studies have looked at overall survival and progression-free survival as measures of treatment success in sarcoma patients who undergo ablation procedures [12, 14–16], to our knowledge, this is the first study to analyse specifically the chemotherapy-free interval as a measure of treatment success in sarcoma patients who undergo ablation procedures.

Our results are consistent with those of Fonck et al. who showed that thermal ablation of pulmonary metastases resulted in a median chemotherapy-free survival of 12.2 months in patients with metastatic colorectal cancer [20]. The chemotherapy-free interval has been evaluated as a measure of treatment success following systemic chemotherapy in other malignancies. The OPTIMOX-1 trial compared continuous oxaliplatin and fluorouracil with a strategy of planned oxaliplatin breaks, but with continuous fluorouracil [21]. The OPTIMOX-2 trial compared the intermittent oxaliplatin strategy of OPTIMOX-1 with a complete chemotherapy-free interval strategy [22]. In both trials, there was no reduction in survival with intermittent therapy, but the results of OPTIMOX-2 showed a trend towards improved survival with maintenance fluorouracil during oxaliplatin breaks [21, 22]. The MRC COIN trial compared continuous oxaliplatin and fluoropyrimidine combination with a strategy of intermittent chemotherapy [10]. The MRC COIN trial failed to show noninferiority of intermittent versus continuous chemotherapy as first-line therapy in advanced colorectal cancer. However, it did show that intermittent chemotherapy was associated with improved quality of life, shortened time on chemotherapy, reduced number of hospital visits, and a minimum difference in overall survival. We expect that the benefits of a chemotherapy-free interval should apply to patients who receive treatment for metastatic sarcoma as they did in patients with advanced colorectal cancer.

A chemotherapy-free interval may be particularly beneficial in younger cancer patients. While sarcomas as a group are rare (accounting for less than 1% of all adult solid malignancies), they are one of the more common types of cancer in patients aged 15–45 [23, 24]. In teenagers and young adults, the side effects of anticancer chemotherapy may occur decades after initial treatment. These late effects, specifically in terms of cardiac toxicity, second malignancies, pulmonary complications, and psychosocial difficulties, are generally different from those seen in younger children and adults. As chemotherapy plays a major role in the treatment of sarcomas, younger patients are potentially exposed to a wide range of chemotherapeutic agents, each with distinct late effects [25]. Minimizing exposure to chemotherapy via a chemotherapy-free interval may have a positive effect on preventing the development of these late effects in younger cancer patients.

Interestingly, our study found that histologic subtype and lack of prior chemotherapy were predictive of the chemotherapy-free interval. Patients with liposarcoma had the longest median systemic chemotherapy-free interval (median not reached) compared to patients with all other histologic subtypes (9.5 months). The extended systemic chemotherapy-free interval seen in patients with liposarcoma may be due to the variable biologic behaviour of this tumour, which ranges from indolent disease to extremely aggressive tumour [26]. Patients with leiomyosarcoma had the shortest median cytotoxic chemotherapy-free interval (28.1 months) compared to patients with all other histologic subtypes (median not reached). The relatively short cytotoxic chemotherapy-free interval seen in patients with leiomyosarcoma may be due to the fact that first-line treatment for recurrent metastatic leiomyosarcoma is cytotoxic chemotherapy [27, 28]. Across all end points (overall survival, the systemic chemotherapy-free interval, and the cytotoxic chemotherapy-free interval), patients who had not failed previous chemotherapy had better outcomes compared to patients who had failed previous chemotherapy. The worse outcomes observed in patients who had failed previous chemotherapy may be explained by a more advanced stage of disease at the time of initial ablation, but it is possible that these patients also had a higher burden of tumour cells that were resistant to chemotherapy [29]. This may have led to the development of more resistant tumours (and worse outcomes) in patients who had failed previous chemotherapy.

This paper has several limitations. First, it is retrospective in nature. Thus, our results may not be applicable to a prospective cohort of sarcoma patients. We had a wide variety of histologic subtypes, and our conclusions may not be applicable to each subtype individually. We had a relatively small number of patients, and our study may be underpowered. Our sarcoma patients were all patients that were referred to the interventional radiology service, and our cohort may not be representative of sarcoma patients more generally. Deaths were not confirmed to be due to the sarcoma itself. Thus, our calculated overall survival may not accurately reflect the actual disease-specific survival. Certain patients had more than one type of cancer, and our conclusions may not be applicable to patients who have only sarcoma. We were not able to assign a grade to every patient's primary sarcoma. Furthermore, since we often did not have pathologic confirmation of grade of the treated metastasis, in some cases its grade was inferred from the grade of the primary tumour. Thus, we could not accurately assess the predictive value of histologic grade. Finally, several new noncytotoxic chemotherapies were approved during the study period and therefore were not available to all patients in the study; this could alter the calculated chemotherapy-free interval. Future prospective studies will be needed to evaluate if image-guided ablation of sarcoma metastases can extend the chemotherapy-free interval in patients with metastatic sarcoma.

## 5. Conclusions

In conclusion, ablation of sarcoma metastases can provide an extended systemic chemotherapy-free interval of greater than 1 year. Ablation of sarcoma metastases may improve patient quality of life by extending the chemotherapy-free interval.

## Figures and Tables

**Table 1:** Demographics and clinical characteristics.

Characteristic	Freq/mean
Sex	
Male	45
Female	55
Age	59 ± 15
>50 years	76
<50 years	24
Tumour size	2.2 ± 1.4
≤3 cm	79
>3 cm	21
Index tumour grade	
High	61
Low	8
Not graded	31
Histologic subtype	
Leiomyosarcoma	38
GIST	14
Liposarcoma	12
Chondrosarcoma	7
Other	29
Failed previous chemotherapy	
No	48
Yes	52
Disease-free interval	
<12 months	68
>12 months	32

**Table 2:** Overall survival in sarcoma patients undergoing ablation.

Variable	Univariate		Multivariate			
	Median (months)	*p*	Model coefficient	95% CI	Hazard ratio	*p*
Sex		0.36				
Male	49.6					
Female	53.1					
Age		0.92				
>50 years	52.4					
<50years	55.9					
Tumour size		0.033	0.54	0.87–3.38	1.72	0.1159
≤3 cm	53.1					
>3 cm	31.6					
Index tumour grade		0.19				
High	53.1					
Low	Not reached					
Histologic subtype		0.17^*∗*^				
Leiomyosarcoma	55.9					
GIST	78.1					
Liposarcoma	Not reached					
Chondrosarcoma	24.3					
Other	46.9					
Failed previous chemotherapy		0.027	−0.56	0.31–1.06	0.57	0.0749
No	62.6					
Yes	33.2					
Disease-free interval		0.87				
<12 months	52.4					
>12 months	55.7					

^*∗*^Log-rank tests of no differences among categories vs. any differences among categories.

**Table 3:** Systemic chemotherapy-free interval in sarcoma patients undergoing ablation.

Variable	Univariate		Multivariate			
	Median (months)	*p*	Model coefficient	95% CI	Hazard ratio	*p*
Sex		0.71				
Male	10.9					
Female	14.7					
Age		0.95				
>50 years	20.1					
<50 years	8.8					
Tumour size		0.19				
≤3 cm	20.1					
>3 cm	6.9					
Index tumour grade		0.44				
High	14.7					
Low	10.9					
Histologic subtype		0.017^*∗*^	–0.03	0.83–1.15	0.97	0.758
Leiomyosarcoma	10.4					
GIST	6.9					
Liposarcoma	Not reached					
Chondrosarcoma	24.5					
Other	9.4					
Failed previous chemotherapy		<0.0001	–1.90	0.08–0.30	0.15	<0.0001
No	66.8					
Yes	6.1					
Disease-free interval		0.29				
<12 months	10.9					
>12 months	46.3					

^*∗*^Log-rank tests of no differences among categories vs. any differences among categories.

**Table 4:** Cytotoxic chemotherapy-free interval in sarcoma patients undergoing ablation.

Variable	Univariate		Multivariate			
	Median (months)	*p*	Model coefficient	95% CI	Hazard ratio	*p*
Sex		0.79				
Male	81.3					
Female	46.7					
Age		0.65				
>50 years	81.3					
<50 years	Not reached					
Tumour size		0.16				
≤3 cm	81.3					
>3 cm	34.3					
Index tumour grade		0.78				
High	Not reached					
Low	81.3					
Histologic subtype		0.034^*∗*^	–0.03	0.78–1.19	0.97	0.751
Leiomyosarcoma	28.1					
GIST	Not reached					
Liposarcoma	Not reached					
Chondrosarcoma	Not reached					
Other	81.3					
Failed previous chemotherapy		<0.0001	−1.92	0.06–0.36	0.15	<0.0001
No	Not reached					
Yes	12.2					
Disease-free interval		0.34				
<12 months	81.3					
>12 months	Not reached					

^*∗*^Log-rank tests of no differences among categories vs. any differences among categories.

## Data Availability

The retrospective data used to support the findings of this study have not been made available because of ownership reasons.
